# Acute kidney injury predicts poor left ventricular function for patients with peripartum cardiomyopathy

**DOI:** 10.1186/s12872-021-02021-6

**Published:** 2021-04-22

**Authors:** Jiajia Zhu, Wenxian Liu

**Affiliations:** Department of Cardiology, Beijing Anzhen Hospital, Capital Medical University, Beijing Institute of Heart, Lung and Blood Vessel Disease, No. 2 Anzhen Road, Beijing, 100029 China

**Keywords:** Acute kidney injury, Left ventricular ejection fraction, Peripartum cardiomyopathy

## Abstract

**Background:**

The aim of this study was to explore the risk factors associated with a poor left ventricular (LV) function among patients with peripartum cardiomyopathy (PPCM) and to determine the influence of acute kidney injury (AKI) on the LV function of the patients.

**Methods:**

Sixty patients with PPCM were recruited between January 2007 and June 2018, among which 11 had AKI. The participants were divided into two groups, the recovery group (32 cases) and the nonrecovery group (28 cases), with their clinical features, echocardiography and electrocardiogram findings, laboratory results, and treatments compared between groups. We further determined the risk factors associated with nonrecovery and the influence posed by AKI on the LV function of the patients.

**Results:**

Compared with the patients in the recovery group, those in the nonrecovery group had higher proportions of multiparity [78.6% (22/28) vs. 43.8% (14/32)], function class III– IV heart failure [92.9% (26/28) vs. 71.9% (23/32)], and a higher incidence of AKI [35.7% (10/28) vs. 3.1% (1/32)]. Logistic regression analysis showed that having AKI [odds ratio (OR): 10.556; 95% confidence interval (CI) 1.177–94.654; *P* = 0.035] and left ventricular ejection fraction (LVEF) < 40% [OR: 4.533; 95% CI 1.118–18.382; *P* = 0.034] were independently associated with nonrecovery of PPCM.

**Conclusions:**

The prognosis of patients with PPCM and AKI during hospitalization was poor compared to those without AKI; therefore, clinicians should pay more attention to this phenomenon.

## Background

Peripartum cardiomyopathy (PPCM) is an idiopathic disease. Patients with PPCM present with clinical symptoms of heart failure secondary to left ventricular (LV) dysfunction towards the end of their pregnancy or during the postpartum period, mostly within months following delivery. PPCM is characterized echocardiographically by the presence of a LV ejection fraction (LVEF) < 45% and/or fractional shortening < 30% [1]. According to prior studies, no other cause of heart failure has been found [1]. As an uncommon and potentially life-threatening illness, PPCM rarely has its cause(s) identified. Several risk factors for PPCM have been reported previously, including an advanced age, ethnicity (especially African), multiple gestations, hypertension during pregnancy, the LVEF and LV end diastolic diameter (LVEDD) at the diagnosis of PPCM, and the presence of LV thrombi [2–4]. However, it remains unclear whether more risk factors exist, and this is still the subject of ongoing debates.

As a complication of heart failure, acute kidney injury (AKI) has been shown to worsen the prognosis of patients with heart failure. Acute deterioration of renal function occurs in 20–40% of patients hospitalized for acute decompensated heart failure (ADHF) [5, 6]. Regardless of the timing of AKI onset, patients with ADHF and renal dysfunction have a significantly greater risk of rehospitalization and all-cause mortality compared to those without renal dysfunction [7]. In a retrospective study involving patients with heart failure, worsening renal function occurred in 11% of patients during hospitalization and in 16% of patients during the first 6 months after discharge [7]. Despite the established negative effects posed by AKI, the prognostic influence of AKI in patients with PPCM has not been described previously. Therefore, the aim of this study was to investigate the clinical features associated with PPCM and to determine the predictive value of in-hospital AKI for nonrecovery of PPCM among affected patients.

## Methods

### Population and study design

A total of 60 patients with PPCM admitted to Beijing Anzhen Hospital Affiliated to Capital Medical University were prospectively enrolled in this study from January 2007 to June 2018. The inclusion criteria were the presence of a PPCM diagnosis according to the PPCM Working Group of the European Society of Cardiology made by cardiologists and obstetricians; heart failure symptoms and a left ventricular ejection fraction (LVEF) below 45% at enrollment; an age of at least 18 years old; a heart failure severity of New York Heart Association (NYHA) functional class II–IV (for new patients only); and written informed consent. Patients were excluded from this study if they had a history of cardiomyopathy or severe organic valvular heart disease, had chronic hypertension, or failed to provide reliable contact phone numbers. The diagnosis of PPCM was made based on the diagnostic criteria endorsed by the European Society of Cardiology [1], including the following three criteria: first, the event occurred during the last month of pregnancy or within months following delivery; second, causes of heart failure other than PPCM could be excluded; finally, the echocardiographic findings of the index patients showed that LV systolic function was significantly impaired, manifesting as LVEF < 45% with or without LV enlargement. The demographic data, clinical features, laboratory and echocardiography parameters, and treatment received for the patients with PPCM were collected from their medical records. AKI was diagnosed according to the diagnostic criteria proposed by the Kidney Disease: Improving Global Outcomes (KDIGO) in 2012. Patients with a serum creatinine level > 1.5 times their baseline value or an increase of ≥ 0.3 mg/dL (≥ 26.5 μmol/L) within 48 h were diagnosed as having AKI [8].

All patients were followed up for at least 6 months after diagnosis. After follow-up, it was determined whether the participants’ PPCM recovered, defined as LV systolic function improvement according to echocardiographic findings of LVEF > 50%. On the contrary, nonrecovery of PPCM was identified if the patients had LVEF ≤ 50%, received a heart transplantation, or died after 6 months of follow-up. The patients with PPCM were divided into the recovery group and the nonrecovery group, with the proportion of patients developing AKI documented. Finally, using logistic regression analysis, whether the presence of AKI was associated with nonrecovery among patients with PPCM was determined.

The Ethics Committee of Beijing Anzhen Hospital approved the current study (Approval No. 2019041X). Written informed consent was obtained from all individual participants included in this study.

### Statistical analysis

All data were analyzed with SPSS 20.0 software (SPSS Inc., Chicago, IL, USA). Continuous variables were expressed as means ± standard deviation, and categorical variables were expressed as percentages. Continuous variables were analyzed with the Student’s t test or the Mann–Whitney U test. Categorical variables were compared using Pearson’s chi-squared test. To assess the relationship between AKI and the prognosis of patients with PPCM, multivariate stepwise logistic regression analyses were conducted. A two-tailed *p* value < 0.05 was considered significant in all analyses.

## Results

Sixty patients with PPCM were enrolled in this study, among whom 11 (18.3%) had AKI. The mean age of these participants was 30 ± 5 years, and 36 (60%) were multiparous. Three (5%) of them had a history of PPCM. PPCM was diagnosed in 38 (63.3%) participants before delivery and in 22 (36.7%) participants during the postpartum period. With regard to their past medical history, 6 (10.0%) and 4 (6.7%) had hypertension and diabetes, respectively, while 12 (20.0%) developed hypertension during their pregnancy, among whom 5 (8.3%) had pregnancy-induced hypertension (PIH) and 7 (11.7%) had preeclampsia. Their mean blood pressure (BP) during hospitalization was 116 ± 18/77 ± 14 mmHg, and their mean heart rate was 102 ± 20 beats per minute (bpm). Forty-four (73.3%) had ST-T changes in their electrocardiograms. Their LVEDD and LVEF values were 59 ± 8 mm and 37 ± 13%, respectively. Five (8.3%) had intra-cardiac thrombosis. With regard to the treatments, all received diuretics, while 40 (66.7%), 42 (70.0%), 11 (18.3%), and 3 (5.0%) received angiotensin-converting enzyme inhibitors/angiotensin receptor blockers (ACEIs/ARBs), β-blockers, anticoagulants, and vasoactive drugs including dopamine, epinephrine, and norepinephrine, respectively. Six (10.0%) were treated with mechanical ventilation, among whom 4 (6.7%) and 2 (3.3%) received noninvasive and invasive ventilation, respectively. One patient was treated with intra-aortic balloon counter-pulsation (IABP) and extracorporeal membrane oxygenation (ECMO) (Table 1).

**Table 1:** Baseline characteristics of patients with PPCM (n = 60)

Characteristic	Value
Age (years)	30 ± 5
Primiparous (%)	24 (40.0)
Prenatal diagnosis (%)	38 (63.3)
Prenatal diagnosis (weeks)	30 ± 11
Postnatal diagnosis (%)	22 (36.7)
Postnatal diagnosis (months)	1 (1–3)
Cesarean section (%)	53 (88.3)
NYHA III–IV (%)	49 (81.7)
Hospitalization time (days)	12 (7–15)
Hypertension (%)	6 (10.0)
Diabetes mellitus (%)	4 (6.7)
PPCM history (%)	3 (5.0)
Pregnancy-induced hypertension syndrome (%)	5 (8.3)
Preeclampsia (%)	7 (11.7)
SBP (mmHg)	116 ± 18
DBP (mmHg)	77 ± 14
HR (bpm)	102 ± 20
Echocardiographic findings	
LVEF (%)	37 ± 13
Left ventricular thrombosis (%)	5 (8.3)
LVEDD (mm)	59 ± 8
Electrocardiographic findings	
ST-T changes (%)	44 (73.3)
QRS time (ms)	94.2 ± 21.5
QTc time (ms)	475 ± 29
Hemoglobin (g/L)	123 ± 16
Platelet (G/L)	262 ± 84
ALT (U/L)	22 (12–46)
AST (U/L)	24 (19–34)
Serum creatinine (μmol/L)	64.2 (54.1–69.3)
BNP (pg/mL)	819 (378–2143)
AKI (%)	11 (18.3)
Ventilator (%)	6 (10.0)
Noninvasive ventilator (%)	4 (6.7)
Invasive mechanical ventilator (%)	2 (3.3)
IABP (%)	1 (1.7)
ECMO (%)	1 (1.7)
CRRT (%)	2 (3.3)
Diuretic (%)	60 (100)
ACEI/ARB (%)	40 (66.7)
β-Blockers (%)	42 (70.0)
Ivabradine (%)	3 (5.0)
Anticoagulation (%)	11 (18.3)
Digoxin (%)	30 (50.0)
Vasoactive agents (%)	3 (5.0)

*NYHA* New York Heart Association, *PPCM* peripartum cardiomyopathy, *SBP* systolic blood pressure, *DBP* diastolic blood pressure, *HR* heart rate, *LVEF* left ventricular ejection fraction, *LVEDD* left ventricular end-diastolic diameter, *ALT* alanine transaminase, *AST* aspartate transaminase, *AKI* acute kidney injury, *IABP* intra-aortic balloon pump, *ECMO* extracorporeal membrane oxygenation, *CRRT* continuous renal replacement therapy, *ACEI/ARB* angiotensin-converting enzyme inhibitor/angiotensin receptor blocker

With regard to outcomes, 32 (53.3%) participants recovered from PPCM, while 28 (46.7%) did not. The mean age of the nonrecovered participants was older (nonrecovery vs. recovery, 32 vs. 29 years, *P* = 0.038) than that of the recovered ones, and the proportion of multiparous participants was higher as well (nonrecovery vs. recovery, 78.6% vs. 43.8%, *P* = 0.006). There was no significant difference between groups regarding the past history of PPCM (nonrecovery vs. recovery, 7.1% vs. 3.1%, *P* = 0.594), hypertension (10.7% vs. 9.4%, *P* = 1.000), diabetes (10.7% vs. 3.1%, *P* = 0.331), or PIH (17.9% vs. 21.9%, *P* = 0.698). The systolic BP was significantly lower in the nonrecovery group (nonrecovery vs. recovery, 111 vs. 122 mmHg, *P* = 0.02), but the heart rates (105 vs. 99 bpm, P = 0.270) were comparable between groups. The nonrecovered participants had a significantly higher LVEDD (nonrecovery vs. recovery, 53 vs. 43 mm, *P* = 0.003) and a significantly lower LVEF (30% vs. 43%, *P* < 0.001) than the recovered ones, while the proportion of participants with intracardiac thrombosis (14.3% vs. 3.1%, *P* = 0.175) or electrocardiographic ST-T changes (78.6% vs. 68.8%, *P* = 0.391) did not differ. Regarding the laboratory tests, the nonrecovered participants had significantly higher serum creatinine (nonrecovery vs. recovery, 66.3 vs. 54.1 μmol/L, *P* < 0.001) and brain natriuretic peptide (BNP) levels (1436 vs. 356 pg/mL, *P* < 0.001) on admission than the recovered ones. During hospitalization, all participants received diuretics. The proportion of digoxin users in the nonrecovery group was higher (75.0% vs. 28.1%, *P* < 0.001) than that in the recovery group, while there was no difference in the use of ACEIs/ARBs (nonrecovery vs. recovery, 75.0% vs. 59.4%, *P* = 0.200), β-blockers (75.0% vs. 65.6%, *P* = 0.429), anticoagulants (21.4% vs. 15.6%, *P* = 0.562), vasoactive drugs (7.1% vs. 3.1%, *P* = 0.594), ventilation (14.3% vs. 6.3%, *P* = 0.404), IABP (3.6% vs. 0%, *P* = 0.467), ECMO (3.6% vs. 0%, *P* = 0.467), or continuous renal replacement therapy (CRRT) (7.1% vs. 0%, *P* = 0.214) between groups (Table 2).

**Table 2:** Comparison of the clinical and laboratory characteristics between the recovery and the nonrecovery groups (n = 60)

Characteristic	Recovery Group (n = 32)	Nonrecovery Group (n = 28)	*P* value
AKI (%)	1 (3.1)	10 (35.7)	0.002
Age (years)	29 ± 4	32 ± 6	0.038
Primiparous (%)	14 (43.8)	22 (78.6)	0.006
Cesarean section (%)	28 (87.5)	28 (100)	0.116
NYHA III–IV (%)	23 (71.9)	26 (92.9)	0.048
Hospitalization time (days)	12 (8–15)	11 (6–15)	0.542
Hypertension (%)	3 (9.4)	3 (10.7)	1.000
Diabetes mellitus (%)	1 (3.1)	3 (10.7)	0.331
PPCM history (%)	1 (3.1)	2 (7.1)	0.594
Pregnancy-induced hypertension syndrome (%)	2 (6.3)	3 (10.7)	0.657
Preeclampsia (%)	5 (15.6)	2 (7.1)	0.432
SBP (mmHg)	122 ± 16	111 ± 17	0.02
DBP (mmHg)	79 ± 11	74 ± 15	0.1
HR (bpm)	99 ± 19	105 ± 20	0.27
Echocardiographic findings			
LVEF (%)	43 ± 12	30 ± 10	< 0.001
Left ventricular thrombosis (%)	1 (3.1)	4 (14.3)	0.175
LVEDD (mm)	43 ± 8	53 ± 8	0.003
Electrocardiographic findings			
ST-T changes (%)	22 (68.8)	22 (78.6)	0.391
QRS time (ms)	94 ± 26	95 ± 14	0.808
QTc time (ms)	463 ± 42	457 ± 39	0.64
Hemoglobin (g/L)	120 ± 13	126 ± 19	0.136
Platelet (G/L)	250 ± 80	276 ± 87	0.25
ALT (U/L)	20 (10–35)	23 (16–63)	0.11
AST (U/L)	22 (16–30)	26 (21–45)	0.069
Serum creatinine (μmol/L)	54.1 (44–65.42)	66.3 (56.87–83.25)	< 0.001
BNP (pg/mL)	356 (67–819)	1436 (696–2353)	< 0.001
Ventilator (%)	2 (6.3)	4 (14.3)	0.404
IABP (%)	0	1 (3.6)	0.467
ECMO (%)	0	1 (3.6)	0.467
CRRT (%)	0	2 (7.1)	0.214
Diuretic (%)	32 (100)	28 (100)	1.000
ACEI/ARB (%)	19 (59.4)	21 (75.0)	0.200
β-blockers (%)	21 (65.6)	21 (75.0)	0.429
Ivabradine (%)	2 (6.3)	1 (3.6)	1.000
Anticoagulation (%)	5 (15.6)	6 (21.4)	0.562
Digoxin (%)	9 (28.1)	21 (75.0)	< 0.001
Vasoactive agents (%)	1 (3.1)	2 (7.1)	0.594

*NYHA* New York Heart Association, *PPCM* peripartum cardiomyopathy, *SBP* systolic blood pressure, *DBP* diastolic blood pressure, *HR* heart rate, *LVEF* left ventricular ejection fraction, *LVEDD* left ventricular end-diastolic diameter, *ALT* alanine transaminase, *AST* aspartate transaminase, *AKI* acute kidney injury, *IABP* intra-aortic balloon pump, *ECMO* extracorporeal membrane oxygenation, *CRRT* continuous renal replacement therapy, *ACEI/ARB* angiotensin-converting enzyme inhibitor/angiotensin receptor blocker

All participants were divided into two groups: those with AKI (AKI group; 11 patients) and those without AKI (non-AKI group; 49 patients), according to the diagnostic criteria during hospitalization. There was no difference between those with and without AKI regarding age (32 vs. 30 years, *P* = 0.356), multiparity (72.7% vs. 57.1%, *P* = 0.34), a past history of PPCM (0% vs. 6.1%, *P* = 1.000), hypertension (0% vs. 12.2%, *P* = 0.221), diabetes (0% vs. 8.2%, *P* = 1.000), PIH (27.3% vs. 18.4%, *P* = 0.677), systolic BP (112 ± 20 vs. 118 ± 17 mmHg, *P* = 0.334), diastolic BP (79 ± 16 vs. 76 ± 13 mmHg, *P* = 0.52), mean heart rate (106 vs. 101 bpm, *P* = 0.408), or ST-T changes (90.9% vs. 69.4%, *P* = 0.259). However, the participants with AKI had a significantly lower LVEF (AKI vs. non-AKI, 29% vs. 38%, *P* = 0.026) and a higher proportion of intra-cardiac thrombosis (27.3% vs. 4.1%, *P* = 0.039) than those without. With regard to the laboratory results, the levels of serum creatinine (79.5 vs. 55.5 μmol/L, *P* < 0.05) and BNP (2243 vs. 546 pg/mL, *P* < 0.05) in the AKI group were significantly greater than those in the non-AKI group on admission. During hospitalization, the proportions of digoxin (90.9% vs. 40.8%, *P* = 0.006) and anticoagulant users (54.5% vs. 10.2%, *P* = 0.001) were greater in the AKI group than in the non-AKI group. Meanwhile, there was no difference between groups regarding the proportion of those requiring ACEI/ARB (63.6% vs. 67.3%, *P* = 1.000), β-blockers (54.5% vs. 73.5%, *P* = 0.216), vasoactive drugs (18.2% vs. 2.0%, *P* = 0.084), ventilation (27.3% vs. 6.1%, *P* = 0.069), IABP (9.1% vs. 0%, *P* = 0.183), or ECMO (9.1% vs. 0%, *P* = 0.183). Two patients with AKI received CRRT (Table 3).

**Table 3:** Comparison of the clinical characteristics between patients with and without AKI (n = 60)

Characteristic	AKI (n = 11)	Non-AKI (n = 49)	*P* value
Age (years)	32 ± 6	30 ± 5	0.356
Primiparous (%)	8 (72.7)	28 (57.1)	0.34
Cesarean section (%)	11 (100.0)	43 (91.8)	1.000
NYHA III–IV (%)	11 (100)	38 (77.6)	0.189
Hospitalization time (days)	14 (8–23)	11 (7–15)	0.105
Hypertension (%)	0	6 (12.2)	0.221
Diabetes mellitus (%)	0	4 (8.2)	1.000
PPCM history (%)	0	3 (6.1)	1.000
Pregnancy-induced hypertension syndrome (%)	0	5 (10.2)	0.573
Preeclampsia (%)	3 (27.3)	4 (8.2)	0.108
SBP (mmHg)	112 ± 20	118 ± 17	0.334
DBP (mmHg)	79 ± 16	76 ± 13	0.52
HR (bpm)	106 ± 19	101 ± 20	0.408
Echocardiographic findings			
LVEF (%)	29 ± 11	38 ± 13	0.026
Left ventricular thrombosis (%)	3 (27.3)	2 (4.1)	0.039
LVEDD (mm)	52 ± 8	47 ± 9	0.386
Electrocardiographic findings			
ST-T changes (%)	10 (90.9)	34 (69.4)	0.259
QRS time (ms)	88 ± 10	96 ± 23	0.397
QTc time (ms)	457 ± 54	460 ± 37	0.803
Hemoglobin (g/L)	126 ± 17	122 ± 16	0.485
Platelet (G/L)	238 ± 82	267 ± 84	0.3
ALT (U/L)	26 (13–83)	21 (12–43)	0.233
AST (U/L)	27 (25.3–29.4)	22 (16–31)	0.097
Serum creatinine (μmol/L)	79.5 (63.4–98.3)	55.5 (52.95–65.75)	0.016
BNP (pg/mL)	2243 (1272–5000)	546.55 (104.63–1403.75)	0.001
Ventilator (%)	3 (27.3)	3 (6.1)	0.069
IABP (%)	1 (9.1)	0	0.183
ECMO (%)	1 (9.1)	0	0.183
CRRT (%)	2 (18.2)	0	0.031
Diuretic (%)	11 (100)	49 (100)	1.000
ACEI/ARB (%)	7 (63.6)	33 (67.3)	1.000
β-blockers (%)	6 (54.5)	36 (73.5)	0.216
Ivabradine (%)	0	3 (6.1)	1.000
Anticoagulation (%)	6 (54.5)	5 (10.2)	0.001
Digoxin (%)	10 (90.9)	20 (40.8)	0.006
Vasoactive agents (%)	2 (18.2)	1 (2.0)	0.084

*NYHA* New York Heart Association, *PPCM* peripartum cardiomyopathy, *SBP* systolic blood pressure, *DBP* diastolic blood pressure, *HR* heart rate, *LVEF* left ventricular ejection fraction, *LVEDD* left ventricular end-diastolic diameter, *ALT* alanine transaminase, *AST* aspartate transaminase, *AKI* acute kidney injury, *IABP* intra-aortic balloon pump, *ECMO* extracorporeal membrane oxygenation, *CRRT* continuous renal replacement therapy, *ACEI/ARB* angiotensin-converting enzyme inhibitor/angiotensin receptor blocker

Finally, logistic regression analysis was used to determine the risk factors associated with nonrecovery among all participants with PPCM. Having AKI [odds ratio (OR): 10.556; 95% confidence interval (CI): 1.177–94.654; *P* = 0.035] or an EF < 40% (OR: 4.533; 95% CI 1.118–18.382; *P* = 0.034) was found to be independently associated with nonrecovery of PPCM (Table 4).

**Table 4:** Univariate and multivariate logistic regression analyses of factors associated with nonrecovered peripartum cardiomyopathy

Parameter	OR	95% CI	*P* value	OR	95% CI	*P* value
AKI	17.222	2.034–145.810	0.009	10.556	1.177–94.654	0.035
NYHA III–IV	5.087	0.995–26.006	0.051			
LVEF < 40%	7.714	2.170–27.419	0.002	4.533	1.118–18.382	0.034
LVEDD > 55 mm	4.714	1.506–14.760	0.008	2.758	0.733–10.377	0.134

*AKI* acute kidney injury, *CI* confidence interval, *LVEF* left ventricular ejection fraction, *OR* odds ratio

## Discussion

In this study, the incidence of AKI among patients with PPCM was found to be 18.3%. In addition, AKI was determined to be an independent risk factor associated with nonrecovery in these patients.

PPCM is a type of cardiomyopathy that mainly occurs late during pregnancy or during the first postpartum month. The clinical manifestations and the principles of management of PPCM are similar to those of dilated cardiomyopathy. However, PPCM is unique because it only involves women during their pregnancy or following delivery. The incidence of PPCM differs between countries. In Haiti, the incidence of PPCM is about 1:300 [2]; while in South Africa and United States, it is approximately 1:1000 [3, 9]. In Japan, the incidence of PPCM is only 1:20,000 [10]. With evolution of the definition of PPCM and the increased awareness of this disease, it is now understood that the complications associated with PPCM are devastating. To the best of our knowledge, there is currently a lack of large cohort studies of PPCM in China. A study involving pregnant women treated at Peking Union Medical College Hospital between January 1995 and December 2014 showed that only one maternal morbidity event was noted per 1067 deliveries [11]. Although PPCM is a rare disease, it poses a serious threat to the health of pregnant women and their newborns. One report has suggested that once pregnant women are diagnosed with PPCM, their mortality rate could be as high as 16% [12–14]. In addition, Karaye et al*.* found that the mortality rate was 18.7% in Nigeria in the largest PPCM cohort studied to date [15, 16]. Therefore, it is necessary to gain more understanding regarding the etiologies and risk factors of mortality for patients with PPCM.

The exact etiology of PPCM is still unclear, but the onset of PPCM may be associated with changes in hormone levels, the hemodynamic status, and metabolic states during the perinatal period [17]. Prior studies have further demonstrated that an older age, multiple pregnancies, multiparity, PIH, preeclampsia, and a low socioeconomic status are all risk factors for developing PPCM [17, 18]. In the current study of 60 patients with PPCM, the mean age at delivery of these women was 30 years old. About 60% of the participants had delivered more than once, and nearly 20% had PIH during pregnancy. Therefore, the clinical features of the patients enrolled in this study are consistent with the findings described in the existing literature.

The prognosis of PPCM varies substantially among affected women. It is generally believed that the LVEF of patients with PPCM will return to normal within 6 months after onset. If their LVEF does not recover 6 months later, it is likely that the cardiac damage has become irreversible in these patients and can culminate in mortality [19]. After follow-up, 46.7% of the patients with PPCM did not recover. The patients with nonrecovered PPCM were older than those who had recovered, consistent with the findings of patients who are susceptible to PPCM development. In addition, the patients with nonrecovered PPCM had a lower systolic BP and LVEF at admission but a higher LVEDD, serum creatinine level, and BNP level than those with recovered PPCM. Moreover, the incidence of AKI was higher in those with nonrecovered PPCM, suggesting that worse outcomes occur more frequently in participants with severe hemodynamic disturbances at admission.

AKI has already been established as a risk factor for a poor prognosis in patients with heart failure. The incidence rate of AKI in patients with ADHF is 20–45%, according to different AKI definitions [8, 20, 21], and the presence of AKI results in a 22% increase of mortality. However, no previous studies have addressed AKI and its influence on patients with PPCM. In this study, the incidence of AKI in patients with PPCM was 18.3% during hospitalization. Furthermore, the clinical features between the patients with and without AKI were similar except for a lower LVEF in those with AKI, indicating that AKI is not related to drug use but to the disease per se. In the existing literature, AKI is an independent risk factor for a poor prognosis of patients with ADHF that is independent of a reduced ejection fraction in patients with HFrEF or those with a mid-range ventricular ejection fraction (HFmrEF) [22]. On the other hand, PPCM is a special disease entity distinct from heart failure, and it deserves separate attention. We found that AKI was an independent risk factor for a poor prognosis, regardless of EF, LVEDD, and BNP based on the logistic regression results. Regarding the relationships between AKI, EF, LVEDD, and BNP, those in the AKI group had a lower EF, a larger LVEDD, and a higher BNP than those without. These findings suggest that those with AKI had deteriorations in cardiac function [23, 24]. We believe that the patients in the AKI group had a lower EF, a larger LVEDD, and a higher BNP level, indicating that the condition of PPCM patients is critical. Therefore, we should pay more attention to the influence of AKI in patients with PPCM.

In addition, we found that the serum creatinine level was 79.5 μmol/L in patients with AKI and 55.5 μmol/L in those without, and the hemoglobin level was 126 ± 17 g/L in those with AKI and 122 ± 16 g/L in those without. Patients diagnosed as having chronic renal insufficiency are more likely to have anemia due to reduced EPO levels. However, this change in hemoglobin levels does not necessarily exist in patients with AKI because PPCM frequently presents as acute heart failure. Meanwhile, patients in the AKI group had a critical clinical condition because of their low EF value, larger LVEDD, and high BNP levels. It is likely that the patients received an increased dose of multiple medications to alleviate symptoms, leading to hemoconcentration and higher hemoglobin levels. In our study, although the patients in the AKI group had increased creatinine and hemoglobin levels, there was no statistical difference in the hemoglobin levels between the two groups. A larger sample size is needed to draw a definite conclusion.

In this study, LVEF < 40% and AKI were both determined to be independent risk factors for nonrecovered PPCM. Similarly, Chapa et al. identified that LVEDD > 60 mm and LVEF < 20% were independent risk factors for nonrecovery of cardiac function in 32 patients with PPCM [25]. Goland et al. also found that LVEF < 25% was predictive of severe cardiovascular events in patients with PPCM [5]. In the PEACE register study [15], the researchers consecutively recruited 244 PPCM patients from 14 sites in Nigeria and applied a structured follow-up for a median of 17 months. They found that a maternal age < 20 years [hazard ratio (HR): 2.40, 95% CI 1.27–4.54], hypotension (HR: 1.87, 95% CI 1.02–3.43), tachycardia (HR: 2.38, 95% CI 1.05–5.43), and LVEF < 25% at baseline (HR: 2.11, 95% CI 1.12–3.95) could predict mortality. These results are consistent with ours. However, their finding that the regular use of beta-blockers at the 6-month follow-up (HR: 0.20, 95% CI 0.09–0.41) was independently associated with a lower mortality risk was not found in our study. This may be related to the fact that beta-blockers were used according to the local guidelines for heart failure and PPCM only when needed. AKI at admission was also determined to be an independent risk factor for a poor prognosis. This is due to the fact that the kidney is susceptible to alterations in the perfusion pressure. For example, when hemodynamic disturbance occurs, the renal blood flow is compromised, followed by a decline in the glomerular filtration rate, renal perfusion, and the creatinine clearance rate. Therefore, more attention should be paid to patients with PPCM when AKI occurs during their hospitalization.

### Limitation

This investigation was a single-center study with a small sample size. The data were retrospectively collected from medical reports, and the echocardiographic findings were provided by different examiners. In addition, some of the participants had incomplete data because of the prolonged follow-up duration. However, with improvements in the diagnostic criteria of PPCM, the incidence of PPCM is expected to increase. In the future, a multicenter study will be needed to analyze the etiology and the risk factors for nonrecovery in these patients.

## Conclusion

The findings of this study suggested that AKI at admission was significantly associated with nonrecovered PPCM. If AKI occurs in patients with PPCM during their hospitalization, they tend to have a poor recovery of cardiac function, leading to long-term adverse outcomes. These changes in renal function were not caused by drug use. Therefore, clinicians should pay more attention to AKI in patients with PPCM.

## Data Availability

The datasets generated and analyzed during the present study are available from the corresponding author on reasonable request.

## Abbreviations

PPCM: Peripartum cardiomyopathy
AKI: Acute kidney injury
NYHA: New York Heart Association
BNP: B-type natriuretic peptide
LVEDD: End-diastolic diameter of the left ventricle
LVEF: Left ventricular ejection fraction
OR: Odds ratio
CI: Confidence interval
LV: Left ventricular
ADHF: Acute decompensated heart failure
PIH: Pregnancy-induced hypertension
BP: Blood pressure
bpm: Beats per minute
ACEIs/ARBs: Angiotensin-converting enzyme inhibitors/angiotensin receptor blockers
IABP: Intra-aortic balloon counter-pulsation
ECMO: Extracorporeal membrane oxygenation
SBP: Systolic blood pressure
DBP: Diastolic blood pressure
HR: Heart rate
ALT: Alanine transaminase
AST: Aspartate transaminase
CRRT: Continuous renal replacement therapy
